# Molecular characterization of *Moniezia denticulata* (Rudolphi, 1810) and its distinction from *M. expansa* infecting sheep and goats raised in the north and north-western regions of India

**DOI:** 10.1017/S003118202300063X

**Published:** 2023-08

**Authors:** Susheel Kumar, Harpreet Kaur

**Affiliations:** Parasitology Laboratory, Department of Zoology, Panjab University, Chandigarh, India

**Keywords:** cestode, domestic ruminants, India, *M. expansa*, *Moniezia denticulata*, parasite

## Abstract

The tapeworms of *Moniezia* spp. are heteroxenous parasites and their adult forms occur in ruminants' alimentary tract. They steal a significant portion of hosts' nourishment initiating monieziasis, thereby inflicting economic losses in animal rearing. Despite their high economic importance, the molecular characterization and taxonomic status of these parasites have remained poorly understood. In the present study, cestodes were isolated from the sheep and goats' intestines and were stained with Gower's carmine. Upon careful evaluation of morphological characters, 2 species *Moniezia denticulata* and *Moniezia expansa* were identified. The genomic DNA was extracted and polymerase chain reaction (PCR) amplified targeting regions of mitochondrial cytochrome c oxidase subunit 1 (*cox1*), small subunit ribosomal RNA (SSU rRNA) and internal transcribed spacer 1–5.8S rRNA (ITS1–5.8S rRNA) genes followed by sequencing. The partial sequences of *cox1*, SSU rRNA and ITS1–5.8S rRNA genes of *M. denticulata* generated in the present study revealed that even though they share high similarities with *M. benedeni* (93.2% *cox1*; 92.6% SSU rRNA; 84.70% ITS1–5.8S rRNA) and *M. expansa* (88.85% *cox1*; 92.27% SSU rRNA; 81.70% ITS1–5.8S rRNA), they are not identical to them. In the maximum likelihood phylogenetic trees, *M. denticulata* and *M. expansa* consistently appeared as distinct species from each other. The high values of pairwise divergence between these 2 species collected in the present study confirmed their separate identity. The present study reports the first molecular characterization of *M. denticulata* with reference to *M. expansa* infecting sheep and goats in India.

## Graphical abstract

**Figure:**
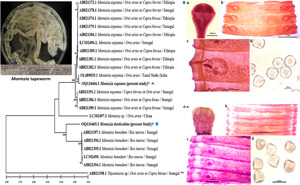

## Introduction

It is well recognized that livestock has many roles in the farm ecosystem and contributes greatly to human livelihoods in developing economies. The small domestic ruminants, sheep and goats, constitute an important part of livestock. They are raised to suffice the need for meat, skin, wool, manure and milk. The incidences of monieziasis, a gastrointestinal disorder, have been a potent cause of declined health of ruminants, thereby inflicting considerable economic losses in the domestic ruminant raising (Bashtar *et al*., 2011; Diop *et al*., 2015; Guo, 2017). The mild pathogenicity of monieziasis is associated with a moderate infection; however, heavy infection often leads to adverse clinical manifestations such as pot-belly, poor growth rate, diarrhoea, anaemia, intestinal pathology, poor quality of wool, fleshless and even death of the ruminant host (Fagbemi and Dipeolu, 1983; Zhao *et al*., 2009; Yan *et al*., 2013). The disease monieziasis is caused by the infection of parasitic anoplocephaline tapeworms of *Moniezia* spp. belonging to the family Anoplocephalidae of the class Cestoda. These tapeworms parasitize the intestine of definitive hosts belonging to the orders Artiodactyla, Perissodactyl and Primates (Ohtori *et al*., 2015). These cestodes show the worldwide distribution and have an indirect mode of life cycle involving oribatid mites as an intermediate host (Nagarajan *et al*., 2022).

In 1891, Blanchard gave the first account of the genus *Moniezia* in which he included 11 species. The concept was elaborated by Moniez in 1878 by including 2 more members in the group (Denegri *et al*., 1998). On the basis of the absence or presence of the interproglottidal glands, Stiles and Hassall (1893) deducted the number of species to 8 and classified them into 3 groups (i) denticulate group without interproglottidal glands, (ii) expansa group comprising sac-like interproglottidal glands and (iii) plannissina group in which the interproglottidal glands show linear arrangement. Baer (1927) in his monographic study of the genus *Moniezia* included 6 species, *M. denticulata* (Rudolphi, 1810), *M*. *expansa* (Moniez, 1810), *M. benedeni* (Moniez, 1879), *M. rugosa* (Diesing, 1810), *M. pallida* (Moning, 1926) and *M. trigonomorpha* (Stiles and Hassall, 1893). The genus *Moniezia* is divided into 3 subgenera in the classical taxonomical scheme proposed by Skrjabin and Schulz in 1937 namely *Moniezia*, *Blanchariezia* and *Baeriezia* differentiated by the presence and type of interproglottidal glands (Haukisalmi *et al*., 2018). In 1982, a new species, *Moniezia sichuanensis* was described from a specimen obtained from wild musk deer. The new species was distinct from the other described species of *Moniezia* by the saw tooth-shaped interproglottidal glands, the thick vagina and the absence of a cirrus spine (Xu *et al*., 2018). The species *M. expansa* is known to infect goats and sheep, *M. benedeni* infects cattle and *M. denticulata* occurs in sheep, goats and cattle.

In India, the incidence of *M. denticulata* infection was recorded by Bhalerao in (1935) from some localities. Saxena and Deo (1964) also gave the morphological account of *M. denticulata* based on the specimen recovered from sheep in Bareilly, India. Other than India, Bashtar *et al*. (2011) reported an 8.5% prevalence of *M. denticulata* in Cairo, Egypt.

In many recent reports on anoplocephalid cestodes, it has been proposed that there is a need of molecular characterization and re-evaluation of the taxonomic status of species (Diop *et al*., 2015; Guo, 2017; Ndom *et al*., 2018, 2019). Despite some recent efforts which have been made to describe the *Moniezia* spp. at the molecular level (Chilton *et al*., 2007; Nguyen *et al*., 2012; Shalaby and Amer, 2012; Yan *et al*., 2013; Ohtori *et al*., 2015; Thosar *et al*., 2015; Kaur and Gupta, 2019; Tam *et al*., 2020; Nagarajan *et al*., 2022), but there is no molecular description of *M. denticulata* available. Although Shalaby and Amer (2012) in their attempt to identify *Moniezia* spp. from Saudi Arabia got some morphological indications of the occurrence of *M. denticulata*, but could not successfully describe it due to lack of molecular data. Therefore, keeping in mind the need for molecular evidence, we provide the first molecular identification and phylogenetic analyses of *M. denticulata* and its comparison with *M. expansa* from the north and north-western region of India based on mitochondrial cytochrome c oxidase subunit 1 (*cox1*) gene, small subunit ribosomal RNA (SSU rRNA) gene and internal transcribed spacer 1–5.8S rRNA (ITS1–5.8S rRNA) gene sequences accompanying the morphological affinities.

## Materials and methods

### Sample collection

In the present study, fresh gut (*n* = 480) of slaughtered sheep (*n* = 67) and goat (*n* = 413) was availed from the abattoir located at Industrial Area, Chandigarh, India where slaughtering is done for consumption purpose. On average, the sheep and goats slaughtered were 2–5 years old. These flocks of sheep and goats were raised in various localities belonging to north-western and northern regions of India. During the period from October 2018 to September 2021, the gut infected with cestodes was carefully transported to laboratory in a biomedical-disposable bag and dissected. The anoplocephalid cestodes were isolated from infected gut and were washed with saline solution (0.85%) to clean. The immature portion of strobila of each worm was cut into small pieces and fixed in 95–99.5% alcohol at −20°C for molecular studies. The remaining portion of worm's body was relaxed in freshly prepared 4% formalin solution (hot). Then, relaxed strobila of each worm was gently intertwined on paper rolls ensuring the morphology was not altered. Subsequent preservation for morphological studies was done by putting the strobila in 4% formalin solution till the use. Out of total infected gut dissected in laboratory, the infection of *M. denticulata* and *M. expansa* was found in 6.7 and 24% gut, respectively. Other guts were having the infection of anoplocephalid tapeworms other than *Moniezia* spp. In total, 16 *M. denticulata* and 51 *M. expansa* were collected.

### Permanent stained preparations and line drawing

The proglottids fixed in 4% formalin were washed in fresh water thoroughly, stained in Gower's carmine (Gower, 1939) for 50–60 min, dehydrated in ascending grades of the alcohol, i.e. 30, 50 and 70% each for about 20 min, respectively, and differentiated in acid ethanol (100 mL of 70% ethanol + 2 mL of concentrated HCl) (Ndom *et al*., 2018). Further dehydration was done in 70, 90 and 100% for 15–20 min each. Xylene was used to clear the specimen for 30 min and clove oil (for 2 h) was also used. Then specimens were mounted in dibutylphthalate polystyrene xylene (DPX), examined and photographed under Jenoptik progress Magnus photographic unit, Radical Stereo Zoom microscope and LAS version V4.1 (Leica Microsystems, CMS GmbH, Wetzlar, Germany) photographic unit. Line drawings of the parasites were made using projection microscope and camera lucida attached to the compound microscope. Measurements were taken in the software namely, LAS V4.1 using the microscope Leica DM3000 (Leica Microsystems) and Radical, ProgRes (JENOPTIK Optical System Cmbh, version 2.10.0.1). The stained proglottids and scolices have been deposited in the museum of the Department of Zoology, Panjab University, Chandigarh, India (Catalogue No: Md/001/m1/2021–22; Md/001/m2/2021–22; Md/001/m3/2021–22; Md/001/m4/2021–22; Me/001/m1/2021–22; Me/001/m2/2021–22; Me/001/m2/2021–22; Me/001/m3/2021–22; Me/001/m4/2021–22).

### DNA isolation, amplification and sequencing

The immature fragments of cestodes, which were fixed in ethanol (95–99.5%) at −20°C immediately following the enough normal saline wash, were used to extract the genomic DNA for molecular analysis. The Qiagen's DNeasy tissue kit was used to isolate the genomic DNA following the tissue DNA extraction protocol prescribed by the manufacturer. To determine the quality and quantity of the extracted genomic DNA, agarose gel electrophoresis and nanodrop spectrophotometer analysis were performed, respectively. The extracted genomic DNA which had desired quality and quantity was used as the template for polymerase chain reactions (PCRs). For the amplification of SSU rDNA gene, primers were designed in the present study; left primer SSU mbF: 5′-ACGGGTCCTTCAAATGTCTG-3′ and right primer SSU mbR: 3′-GGTCTGACTCGTTGACAC-5′ with the aid of Integrated DNA Technologies's (IDT's) oligo analyser tool (https://sg.idtdna.com/pages/tools/oligoanalyzer) and Primer 3 software (Untergasser *et al*., 2012) using the SSU rDNA gene sequences of *Moniezia* spp. available in the National Centre for Biotechnology Information (NCBI). The newly designed pair of primes yielded the PCR product which had a size of 1893 bp. To amplify a portion of *cox1* gene, the left and right primers MoCox1F (5′-CTGAGTGTTTTCAAAACATTTAG-3′) and MoCox1R (5′-AAGCATGATGCAAAAGGCA-3′) (Diop *et al*., 2015), respectively, were referred. The amplification of the ITS1–5.8S region of ribosomal DNA was achieved by using up the left primer ITS1-F (5′-GCTGCTACCCGCATGATGTT-3′) and the right primer ITS1-R (5′-GGCAAGCCTATAGCCGCAAT-3′) (Nguyen *et al*., 2012) for conventional PCRs.

PCRs were carried out in a reaction mixture having a total volume of 25 *μ*L containing 2.5 *μ*L 10× PCR buffer including 2 mm MgCl_2_, 2 *μ*L of each primer of 10 *μ*m concentration (20 pmol of each primer in final volume of reaction mixture), 2 *μ*L of deoxynucleotide triphosphates (dNTPs) of 0.2 mm, 2.5 *μ*L DNA template, 0.6 units of Taq polymerase and 13.8 *μ*L molecular grade nuclease-free water. PCR amplification's 35 cycles, having steps of denaturation (at 94°C) for 30 s, annealing (at 55.7°C for cox1 gene, 57.3°C for SSU rRNA gene and ITS1–5.8s rRNA gene) for 30 s and extension (at 72°C) for 60 s (90 s for SSU rRNA gene), were preceded by an initial denaturation step at 94°C for 5 min. A final extension was carried at 72° C for 5 min. PCR products were visualized by 1.7% agarose gel electrophoresis. Amplified products of expected size were purified by gel extraction and sequences were determined by chain termination method (Sanger *et al*., 1977). Internal primers, designed in the present study, MbF: 5′-TGCGGGACTCATTTAAGAGG-3′ and MbIR: 3′-AACTACCACCACGGATCGTC-5′ were also used to sequence the amplicons of SSU rRNA gene. The sequences were assembled, inspected for vague bases and contigs were generated from the multiple sequences for each molecular marker in BioEdit and manually. The sequences determined in the study, for each molecular marker, were deposited into GenBank of NCBI and accession numbers assigned to sequences were obtained. For the phylogenetic analyses, the sequences of the genus *Moniezia* available in NCBI, for each molecular marker in the present study, were downloaded along with other comparable sequences of closely related species. Multiple sequence alignments of respective molecular marker datasets were carried out using MUSCLE (Multiple Sequence Comparison by Log-Expectation) program in MEGA 11(Tamura *et al*., 2021) with parameters set to default and the alignments were further inspected for any non-conformity and were manually corrected, when needed. Maximum likelihood (ML) phylogenetic trees for sequences of each molecular marker were rebuilt using the best-fit model stated in results. The bootstrapping method, with 1000 replicates, was used to test the robustness of the trees.

## Results

### Morphological analysis of *M. denticulata*

**Figure 1.**
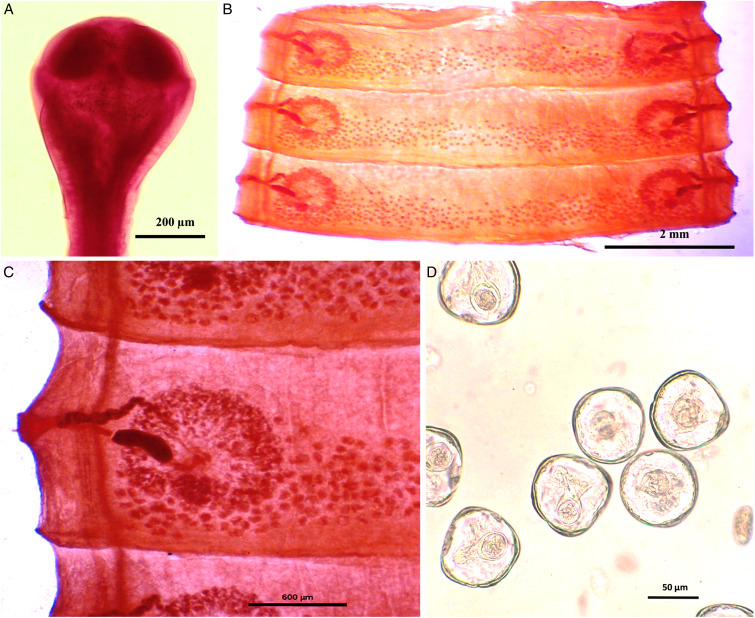
Photomicrograph of *Moniezia denticulata.* (A) Scolex, (B) mature proglottids, (C) magnified portion of a mature proglottid showing morphological details, (D) eggs.

**Figure 2.**
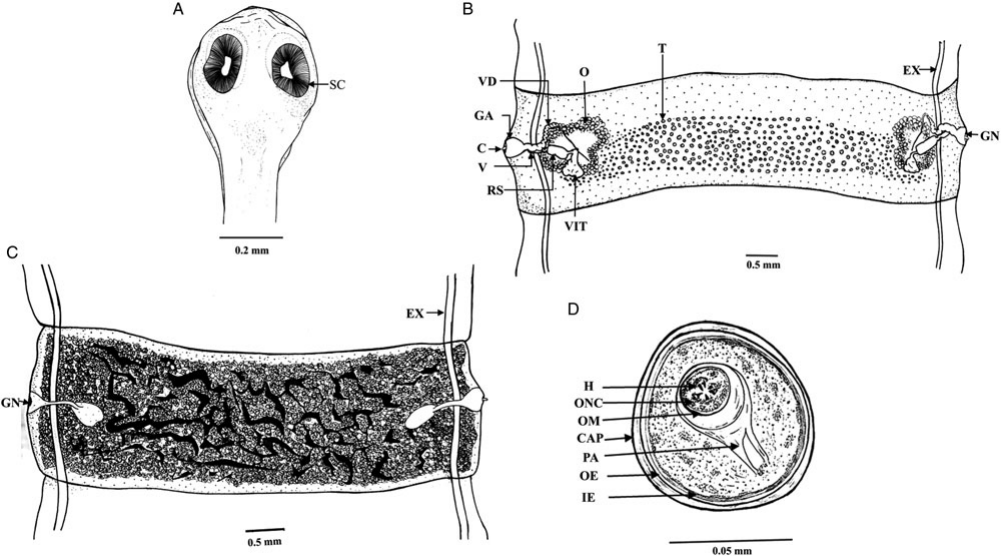
Line drawings of *Moniezia denticulata.* (A) Scolex, (B) mature proglottid, (C) gravid proglottid, (D) egg. O, ovary; T, testis; EX, excretory canal; GN, gonopore; SC, sucker; C, cirrus; V, vagina; RS, receptaculum seminis; GA, genital atrium; VD, vas deference; H, hooks; ONC, onchosphere; OM, onchosphere membrane; CAP, capsule; PA, pyriform apparatus; OE, outer envelope; IE, inner envelope.

Worms whitish in appearance, 3–3.5 m in length and bulky strobila with numerous immature, mature and gravid proglottids (Figs 1A–D and 2A–D; Table 1). Scolex small, spherical, distinguishable from neck, measuring 590–621 (525.8) × 538.3–558 (538.1) *μ*m, unarmed, 4 suckers of dimension 183–222.5 (205.9) × 172–177 (174.7) *μ*m. Scolex followed by short neck which is continuous with strobila. Strobila craspedote but vellum indistinguishable, immature proglottids begin from the neck, 164–294 (229) × 2390–2995 (2692.5) *μ*m, mature proglottids 6–11 times broader than long, 1409.5–1594 (1523.7) × 8901–9225 (9023.2) *μ*m.

**Table 1.** Morphometric comparison of *Moniezia denticulata* and *Moniezia expansa*

Characters	*Moniezia denticulata* (present study)	*Moniezia expansa* (present study)
Scolex (*μ*m)	590–621(525.8) × 538.3–558 (538.1)	523–565 (551.6) × 580–625 (613.8)
Sucker (*μ*m)	183–222.5 (205.9) × 172–177 (174.7)	222–255 (236.8) × 178–202 (192.2)
Immature proglottids	164–294 (229) × 2390–2995 (2692.5)	160–258 (203.2) × 2105–2916 (2590.4)
Mature proglottids (*μ*m)	1409.5–1594 (1523.7) × 8901–9225 (9023.2)	622–692 (674.2) × 3998–4201 (4072.8)
Gravid proglottids (mm)	0.7–1 (0.8) × 1.1–1.8 (1.5)	0.4–0.9 (0.7) × 0.9–1.7 (1.3)
Genital atrium (*μ*m)	21.95–173.8 (139) × 107.4–253.4 (167.3)	19.2–160.5 (89.9) × 101.3–247.6 (174.5)
Cirrus pouch (*μ*m)	169–195 (174.3) × 199.5–252 (226.4)	166–176.5 (168.5) × 199–202.6 (300.7)
Testes (diameter) (*μ*m)	62.2–90.1 (76.15)	32.8–48 (40.4)
Vas deferens (*μ*m)	215–309 (254.1)	260–308 (294)
Cirrus (*μ*m)	43–67 (57.4) × 18–34 (23.8)	53–62.1 (57.5) × 34–53 (47.1)
Interproglottidal glands (*μ*m)	Absent	45–55.5 (50.3) × 42–79.2 (60.6)
Receptaculum seminis (*μ*m)	418–421 (418.1) × 139–141. (139.3)	80.5–-90.1 (85.8) × 50.3–70.6 (60.45)
Vagina (*μ*m)	362.2–371.6 (367.2) × 21.9–24.92 (23.45)	532–567 (172) × 40.1–57.6 (46.86)
Ovary (diameter) (*μ*m)	981.4–1001.7 (993.3)	495–538 (513) × 292–372 (335.16)
Osmoregulatory canal (*μ*m)	120–240 (180)	392–452 (410.4)
Egg (diameter) (*μ*m)	68.01–75.2 (71.6) × 69–78.1 (74.2)	39.01–51.2 (48) × 47–59 (54.5)

Mature proglottids with double male and female reproductive sets, 290–340 (310) testes scattered in posterior half of medulla of proglottids, nearly round in shape, measuring 62.2–90.1 (76.15) *μ*m in diameter. Cirrus sac 169–195 (174.3) × 199.5–252 (226.4) *μ*m, anterior to the vagina, located near to lateral margin running through cup-shaped genital atrium 21.95–173.8 (139) × 107.4–253.4 (167.3) *μ*m. Vas deferens narrow tube, convoluted having diameter of 215–309 (254.1) *μ*m. Cirrus slender, either somewhat curved or straight, protruding out occasionally, 43–67 (57.4) × 18–34 (23.8) *μ*m.

Each female reproductive set includes 1 ovary 981.4–1001.7 (993.3) *μ*m, a prominent receptaculum seminis 418–421 (418.1) × 139–141 (139.3) *μ*m, a ductus vagina 362.2–371.6 (367.2) × 21.9–24.92 (23.45) *μ*m. Vitellaria lobate, 99–109 (104) × 106–124 (115.12) *μ*m, uterus persistent, saccate or reticulate. Interproglottidal glands absent in all proglottids, common genital pores 126–199 (155.76) *μ*m, occurring in genital atrium situated at the middle of each lateral margins. Most of the internal organs disintegrate partially or fully in gravid proglottids. Longitudinal osmoregulatory excretory canals run through the entire length of strobila, occur on either side of the proglottids, narrow, 120–240 (180) *μ*m diameter. Eggs cuboidal, about 68.01–75.2 (71.6) × 69–78.1 (74.2) *μ*m, with thick capsule, contains an embryo, oncosphere with embryonic hooks 2.5–6 (4.25) *μ*m, pyriform apparatus ending in disc.

### Morphological analysis of *M. expansa*

**Figure 3.**
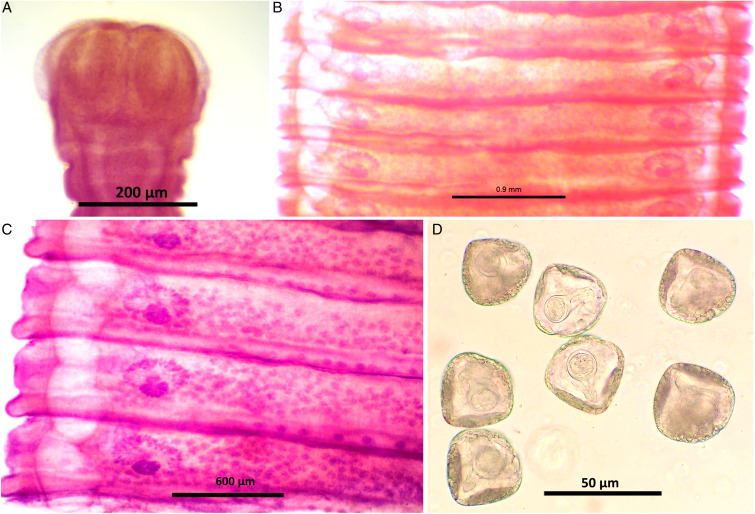
Stained whole mount of *Moniezia expansa*. (A) Scolex, (B) mature proglottids, (C) magnified view showing morphological details of a mature proglottid, (D) eggs.

**Figure 4.**
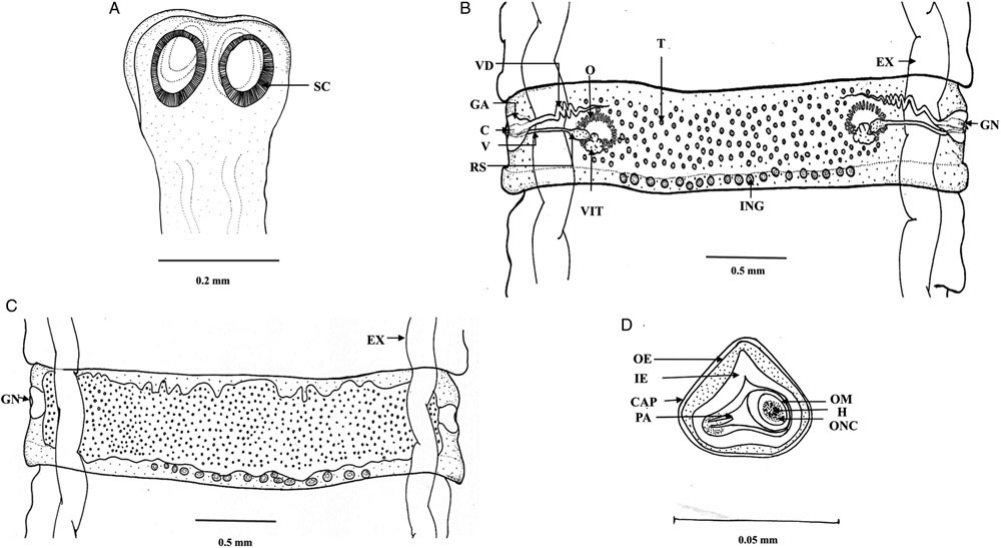
Line diagrams showing the morphological details of *Moniezia expansa*. (A) Scolex, (B) mature proglottid, (C) gravid proglottid, (D) egg. O, ovary; T, testis; EX, excretory canal; GN, gonopore; SC, sucker; C, cirrus; V, vagina; RS, receptaculum seminis; GA, genital atrium; ING, interproglottidal glands; VD, vas deference; H, hooks; ONC, onchosphere; OM, onchosphere membrane; CAP, capsule; PA, pyriform apparatus; OE, outer envelope; IE, inner envelope.

Worms milky white, 2–3.5 m in length, bulky strobila with numerous immature, mature and gravid proglottids (Figs 3A–D and 4A–D; Table 1). Scolex small, distinguishable but not demarcating very clearly from neck, measuring 523–565 (551.6) × 580–625 (613.8) *μ*m, unarmed, 4 wide suckers 222–255 (236.8) × 178–202 (192.2) *μ*m. Short neck 287–312 (295.6) *μ*m following the scolex, not marking off sharply from the strobila. Strobila craspedote with very prominent vellum, mature proglottids 10–11 times broader than long, 622–692 (674.2) × 3998–4201 (4072.8) *μ*m, immature proglottids begin from the neck, 160–258 (203.2) × 2105–2916 (2590.4) *μ*m.

Double reproductive set, 210–260 (235) testes scattered in most of the medullary region of mature proglottids, nearly round in shape, measuring 32.8–48 (40.4) *μ*m. Cirrus pouch measuring 166–176.5 (168.5) × 199–202.6 (300.7) *μ*m, cylindrical, running through genital atrium, located anterior to the vagina. Genital atrium, cup-shaped, 19.2–160.5 (89.9) × 101.3–247.6 (174.5) *μ*m. Vas deferens narrow tube, convoluted having diameter 260–308 (294) *μ*m. Cirrus very slender, slightly curved or straight, 53–62.1 (57.5) × 34–53 (47.1) *μ*m.

Female reproductive set with 2 ovaries of 495–538 (513) × 292–372 (335.16) *μ*m, a receptaculum seminis 80.5–90.1 (85.8) × 50.3–70.6 (60.45) *μ*m, a narrow ductus vagina measuring 532–567 (172) × 40.1–57.6 (46.86) *μ*m, uterus persistent reticulate. Interproglottidal glands present in all proglottids, rosette-like, arranged in a linear row, 18–29 (23) in number, measuring 45–55.5 (50.3) × 42–79.2 (60.6) *μ*m. Vitelaria lobate, 98–103 (98.5) × 97–114 (103.25) *μ*m, common genital pores 116–189 (145) *μ*m situated at the middle of each lateral margins. Longitudinal osmoregulatory excretory canals wide, twisted, occur on either sides of the proglottids, run through the entire length of strobila, measuring 392–452 (410.4) *μ*m in diameter. Eggs nearly triangular or pear-shaped measuring 39.01–51.2 (48) × 47–59 (54.5) *μ*m with thick capsule, contains an oncosphere, embryonic hooks 2.3–6 (4.2) *μ*m, pyriform apparatus present and ends in disc.

### Molecular and phylogenetic analysis

**Figure 5.**
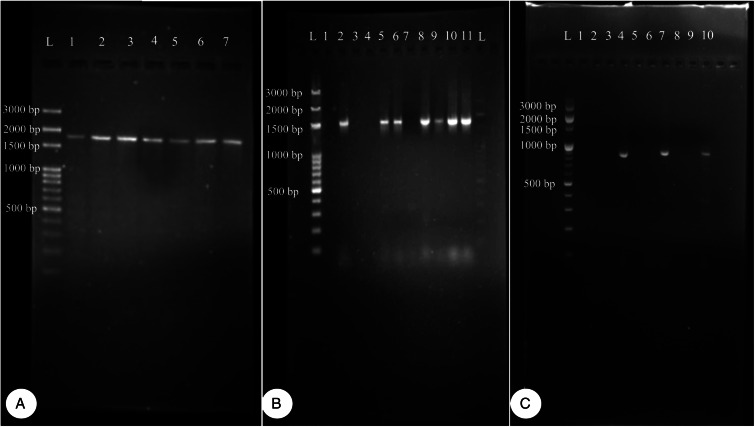
Agarose gel electrophoresis showing the PCR amplifications. (A) SSU rRNA gene (lanes 1–4: *M. denticulata*; lanes 5–7: *M. expansa*). (B) *cox1* gene (lanes 2, 5 and 6: *M. denticulata*; lanes 8–11: *M. expansa*). (C) ITS1–5.8S rRNA gene (lanes 4 and 7: *M. denticulata*; lane 10: *M. expansa*). Letter ‘L’ represents DNA ladder, i.e. size marker.

The primers employed in the present study for the PCR amplification of SSU rDNA gene successfully amplified the amplicons having a size of about 1893 bp (Fig. 5A) and the same were sequenced by employing internal primers in addition to those primers which were used for PCR amplification. After processing, the final sequence of size 1751 bp (*M. expansa*) and 1816 bp (*M. denticulata*) was deposited in NCBI GenBank and accession numbers OM296991.1 (*M. expansa*) and OM296990.1 (*M. denticulata*) were obtained.

The primers referred for *cox1* and ITS1–5.8S rRNA gene yielded the amplicons of size about 1596 bp (Fig. 5B) and 749 bp (Fig. 5C), respectively. After sequencing and processing, the final sequences for *cox1* and ITS1–5.8S rRNA gene of *M. expansa* and *M. denticulata* have sizes of 1574 bp (accession number: OQ134466.1) and 1565 bp (accession number: OQ134465) and 731 bp (accession number: OQ133455.1) and 730 bp (accession number: OQ133454.1), respectively.

The sequences of *cox1*, SSU rRNA, ITS1–5.8S rRNA genes of *M. denticulata* generated in the present study showed 88.85, 92.27 and 81.70% similarity with *cox1*, SSU rRNA, ITS1–5.8S rRNA gene sequence of *M. expansa* (accession no. OQ134466.1) generated in the same study with query coverage of 98, 92 and 78%, respectively, and, 93.2, 92.6 and 84.70% similarity with *M. benedeni* sequences present on online database of NCBI with more than 99% query coverage. The sequence of *M. expansa* generated for *cox1*, ITS1–5.8S rRNA and SSU rRNA gene in the present study showed more than 98% similarity (query coverage more than 99%) with the respective gene sequences of *M. expansa* available on online database of NCBI. To conduct the phylogenetic analyses, the sequences generated in the present study and comparable sequences available from NCBI GenBank for respective molecular markers (*cox1*, SSU rRNA gene, ITS1–5.8S rRNA gene) were used. In phylogenetic analysis, the sequences downloaded from NCBI GenBank and sequence generated in the present study were aligned and non-comparable sequences flanking the sequences of molecular marker under investigation were removed. The ML trees were constructed using 1000 bootstrap replications and Kimura 2-parameter model (Kimura, 1980) with G + I (*γ* distributed with Invariant sites) rate and pattern parameter, and all positions containing gaps and missing data were subjected to partial deletion. The phylogenetic analysis of *cox1* consisted of 22 nucleotide sequences and there were a total of 1590 positions in the final dataset. The codon positions included were 1st + 2nd + 3rd + non-coding, and an ML tree with the highest log-likelihood value of −5560.70 was generated.

**Figure 6.**
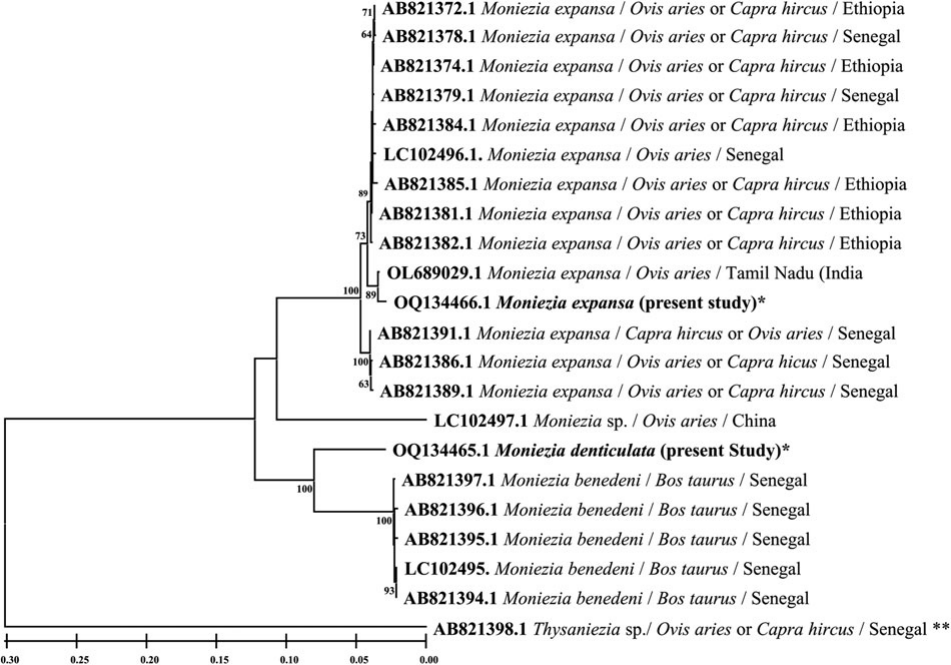
Phylogenetic tree of *Moniezia* spp. using *cox1* gene sequences and generated by using the maximum likelihood (ML) method. Numbers preceding the generic name are accession numbers in the GenBank database and the values of each node are bootstrap percentages (bootstrap percentages above 50% are displayed). **Out-group; *species isolated in the present study.

In the ML tree of *cox1* gene sequences (Fig. 6), the morphotype identified as *M. expansa* in the present study nested within the clade formed by sequences of *M. expansa* deposited in GenBank from various regions of the world, while the sequence of morphotype identified as *M. denticulata* formed a distinct branch with the clade of sequences of *M. benedeni* available from GenBank. The number of base substitutions per site between sequences of *M. denticulata* and *M.* expansa was high ranging from 0.12 to 0.13 and the same ranged from 0.7 to 0.8 between *M. denticulata* and *M. benedeni* suggesting closer relationship but distinct taxa. The overall rates of various transitional and transversionsal base substitutions were 17.37 and 3.82, respectively, with estimated 2.28 transition/transversion bias (*R*).

**Figure 7.**
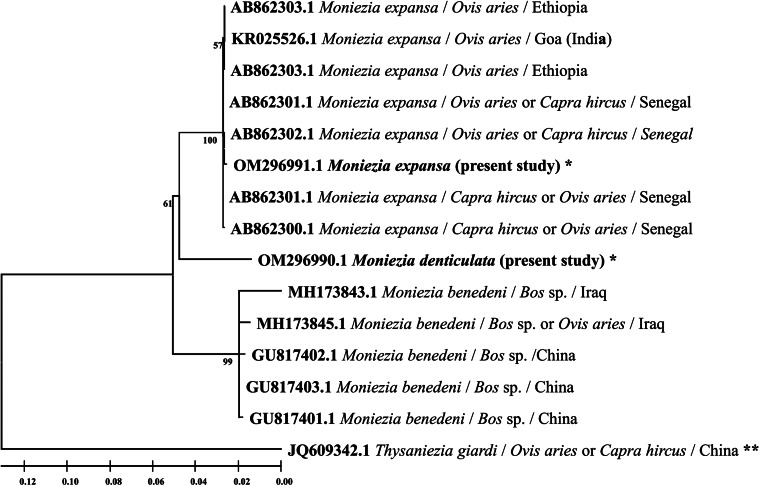
Maximum likelihood (ML) phylogenetic tree of *Moniezia* spp. built using SSU rRNA gene sequences. Numbers preceding the generic name are accession numbers in the GenBank database and the values of each node are bootstrap percentages (bootstrap percentages above 50% are displayed). **Out-group; *species isolated in the present study.

The ML tree, computed including 15 SSU rRNA gene sequences having 1913 positions in the final dataset with highest log-likelihood value −4675.45, showed similar pattern wherein *M. denticulata* formed a distinct branch (Fig. 7), but with the clade formed by *M. expansa* sequences. The number of base substitutions per site between sequences of *M. denticulata* and *M. expansa* was 0.05 and 0.07–0.08 between *M. denticulata* and *M. benedeni.* The rates of various transitional and transversionsal base substitutions were 16.55 and 4.23, respectively, and estimated transition/transversion bias (*R*) was 1.96.

**Figure 8.**
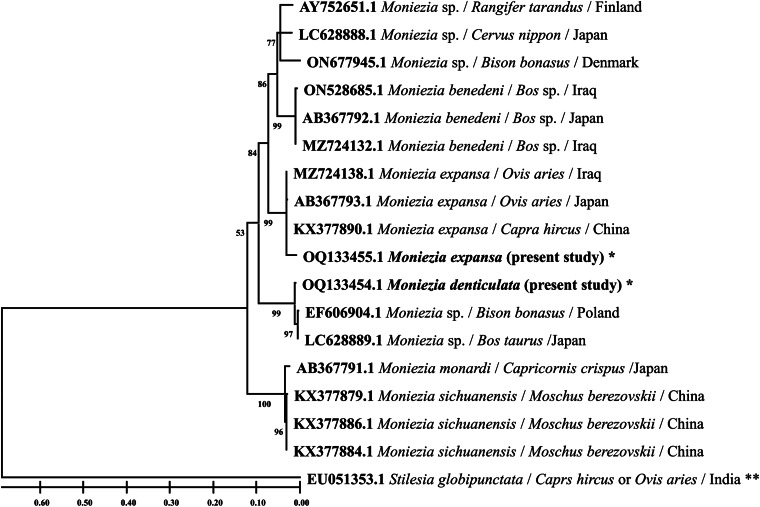
ITS1–5.8S rRNA gene sequence-based phylogenetic tree of *Moniezia* spp. generated by the maximum likelihood (ML) method. Numbers preceding the generic name are accession numbers in the GenBank database and the values of each node are bootstrap percentages (bootstrap percentages above 50% are displayed). **Out-group; *species isolated in the present study.

In the evolutionary analysis based on ITS1–5.8S rRNA nucleotide sequences, the ML tree (Fig. 8) having highest log-likelihood value of −2281.86 was generated by including 18 nucleotide sequences with a total of 510 positions in the final dataset. The number of base substitutions per site between sequences of *M. denticulata* and *M. expansa* ranged from 0.14 to 18 and 0.15 to 0.16 between *M. denticulata* and *M. benedeni.* The rates of various transitional and transversionsal base substitutions were 18.80 and 3.10, respectively, and estimated transition/transversion bias (*R*) was 3.03.

**Figure 9.**
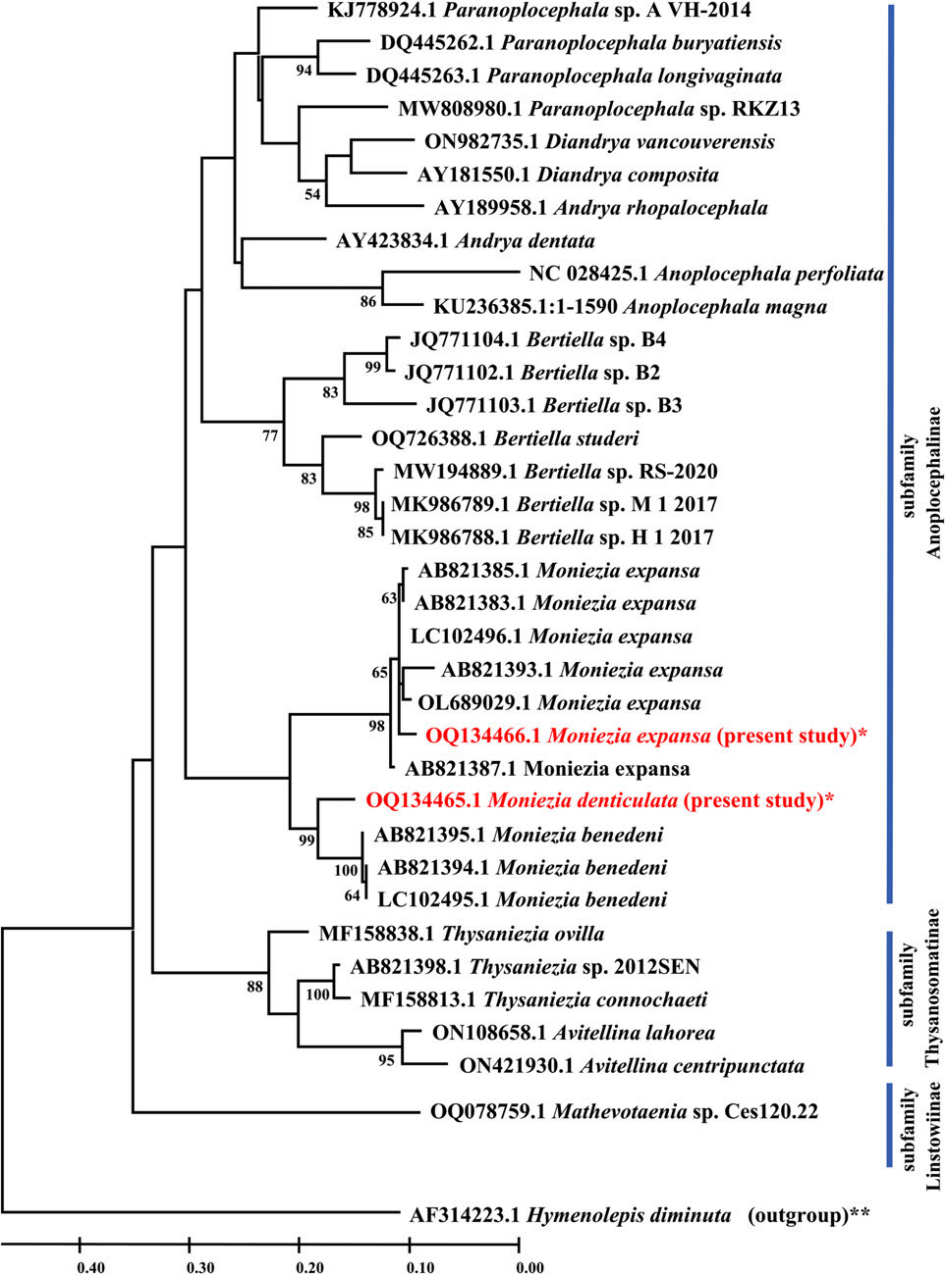
ML phylogenetic tree of anoplocephalid tapeworms showing evolutionary relationship of *Moniezia* spp. with other related genera. The tree was built using *cox1* gene sequences. Numbers preceding the generic name are accession numbers in the GenBank database and the values of each node are bootstrap percentages (bootstrap percentages above 50% are displayed). **Out-group; *species isolated in the present study.

An ML phylogenetic tree (Fig. 9) of *Moniezia* spp. and other anoplocephalid genera was also built using *cox1* genes sequences available from NCBI database and *cox1* gene sequences of *Moniezia* spp. generated in the present study. This analysis involved 36 nucleotide sequences. All positions containing gaps and missing data were eliminated. There were a total of 323 positions in the final dataset.

## Discussion

The tapeworms of *Moniezia* spp. are very common gastrointestinal helminth parasites in domestic ruminants and have exceptionally bulky strobila reaching up to 4 m in length. The continuously proliferating neck adds new proglottids to the ever increasing strobila, and for this, it needs continuous supply of nutrients. To meet its demand for nutrients, it steels host's food and certainly deprives the host from much needed nutrient. Till now, as many as 12 species have been reported from various hosts (Ohtori *et al*., 2015), and in domestic ruminants, only 2 species *M. benedeni* and *M expansa* have been described widely at molecular level. To date, the molecular description and validation of *M. denticulata* has remained very obscure.

In the present study, the description of 2 anoplocephaline cestodes, *M. denticulata* and *M. expansa* isolated from sheep and goats originating from various areas of north and north-western India, is attempted at the molecular level using phylogenetic approach. Upon careful evaluation of morphological characters, 2 types of materials (morphotype) were identified in the present study. One morphotype, which showed the presence of a row of rosette-like interproglottidal glands along the posterior margin of proglottids, was identified as *M. expansa* and the other morphotype in which interproglottidal glands were completely absent throughout the strobila was identified as *M. denticulata*. Other evident differences in morphological characters were the uniformly scattered testes anterior and posterior to ovary in parenchyma of the specimens which possessed rosette-type interproglottidal glands. The testes were distributed in posterior half of proglottids of the specimen which showed complete lacking of interproglottidal glands. The receptaculum semini were more prominent, scolex very distinguishable advocating clear demarcation from neck and even though proglottids were craspetode, vellum was hardly visible in the proglottids of the specimen who lacked interproglottidal glands. Similarly, there were marked differences in the osmoregulatory canals which were wider and twisted in the specimens possessing interproglottidal glands. Besides the structural differences, there were morphometric differences as well (Table 1). The eggs also showed the dissimilarities; they were triangular or pyriform and smaller [39.01–51.2 (48) × 47–59 (54.5) *μ*m] in the case of specimen possessing interproglottidal glands and cuboidal and larger [68.01–75.2 (71.6) × 69–78.1 (74.2) *μ*m] in the case of specimen lacking interproglottidal glands. The receptaculum seminis were more prominent in the specimens without interproglottidal glands. These dissimilarities depict evolutionary events from ancestral condition to the derived condition. The present results contradict the assumption of Chilton *et al*. (2007), inferred from multi-enzyme electrophoresis study, that the specimens of *Moniezia* with or without interproglottidal glands are same. They depicted that the specimens lacking the interproglottidal glands (designated as ‘unknown’ in their study) shared alleles at 13 loci with *M. benedeni* (designated as ‘Bt6–9’ in their study) and 15 loci with *M. expansa* (designated as ‘Ov2–4’ in their study). While questioning the reliability of interproglottidal glands as a diagnostic character, they suggested assigning of the individuals without interproglottidal glands to one of the morphospecies (Bt6-9 or Ov2-4). However, in the present study, it was observed that the conditions of the absence and presence of interproglottidal glands showed synchrony with other features such as difference in egg shape, pattern of testes distribution, etc., as discussed earlier. The differences were also reflected in molecular results as discussed ahead. The present observations are in agreement with the key to the species of *Moniezia* furnished by Bhalerao (1935) in a monographic record of helminth parasites from India, wherein he depicted *Moniezia* tapeworms without interproglottidal glands, with linear interproglottidal glands and with saccular or rosette interproglottidal glands as *M. denticulata*, *M. benedeni* and *M. expansa*, respectively. Similarly, Saxena and Deo (1964) recorded the occurrence of *M. denticulata* in sheep from Bareilly, India, during a survey and mentioned that its eggs were cuboidal and larger than the eggs of *M. expansa*, and interproglottidal glands were lacking. The same are in agreement with the findings of the present study. The monographic study done by Baer (1927) also mentioned the *M. denticulata* as one of the species of the genus *Moniezia* (Denegri *et al*., 1998).

The molecular analysis of the present study revealed that even though sequences of *M. denticulata* share high similarities with *M. benedeni* (93.2% *cox1*; 92.6% SSU rRNA; 84.70% ITS1–5.8S rRNA) and *M. expansa* (88.85% *cox1*; 92.27% SSU rRNA; 81.70% ITS1–5.8S rRNA), they certainly are not identical to sequences of either species. The phylogeny of *cox1* gene clearly stated that *M. denticulata* is a distinct lineage existing somewhere between the closely related *M. expansa* and *M. benedeni* lineages. The high values of pairwise divergence between *M. denticulata* and *M. expansa* (12–13%) collected in the present study depict that they are different taxa of the same genus and this disparity is comparable to the genetic divergence between *M. expansa* and *M. benedeni* (12.8–13.2%) reported by Diop *et al*. (2015). Furthermore, Ndom *et al*. (2018a) recorded 2.9% maximum pairwise divergence between sequences of SSU rDNA of 2 taxonomically valid species of anoplocephalid cestodes namely *Thysaniezia ovilla* and *Thysaniezia connochaeti*, which is again comparable even less than the values of maximum pairwise divergence in SSU rDNA (5% between *M. denticulata* and *M.* expansa; 7–8% between *M. denticulata* and *M. benedeni*) computed in the present study. To confirm the outcomes of *cox1* and SSU rDNA, third molecular marker ITS1–5.8S rDNA was also employed in the present study. The values of pairwise divergence involving ITS1–5.8S rDNA sequences were also very high; 14–18% between *M. denticulata* and *M.* expansa and 15–16% between *M. denticulata* and *M. benedeni* suggesting that the variations were not intraspecific but interspecific.

The ML trees of SSU rRNA and ITS1–5.8S rRNA genes confirmed the phylogenetic differences between *M. denticulata* and *M. expansa* found in the present study. However, there were some variations in topology among ML trees of cox1, SSU rRNA and ITS1–5.8S rRNA genes which could be attributed to the fact that some of the species of *Moniezia* included in the analysis do not have comparable sequences on online database of NCBI for all 3 molecular markers employed in the present investigation. But *M. denticulata* consistently appeared as a distinct taxon from *M. benedeni* and *M. expansa* in all the cases. Apparently, from the topology of these evolutionary trees, the taxonomical validity is assured and the position of *M. denticulata* may be deemed to be a connecting lineage infecting the ovine, caprine and bovine hosts.

In conclusion, the molecular phylogeny and morphological results of the present study profoundly assert that *M. denticulata* is a taxonomically valid species and certainly exhibits very close sister relationship with the species *M. expansa* and *M. benedeni* infecting the sheep and goats and cattle, respectively. The genetic disparity involving partial sequences of 3 molecular markers (*cox1*, SSU rDNA and ITS1–5.8S rDNA) was consistently observed between the 2 anoplocephaline species collected in the present study. In the phylogenetic analysis, these tapeworms clearly appeared as distinct species within the same genus. Undoubtedly, molecular markers are a powerful tool for distinguishing closely resembling individuals or isolates with overlapping morphological features, but the careful evaluation of morphological characters revealed disparity between the cestodes identified as *M. denticulata* and *M. expansa* in the present study advocating that they are separate species, as traditionally acknowledged, of the same genus. Meanwhile, the phylogenetic trees and morphological observations show their clear genetic distinctiveness, their mode of parasitizing domestic ruminants also suggests that these species may have diverged as a consequence of a transfer from caprine and ovine to bovine or vice versa. The present study is the first report on molecular characterization of *M. denticulata* with reference to *M. expansa* infecting sheep and goats raised in north and north-western regions of India. The study would certainly, directly or indirectly, facilitate future identification of these species and improved control of monieziasis.

## Data Availability

The dataset generated in this study is available from corresponding author upon reasonable request.
